# A hemangioma of the sigmoid colon mesentery presenting as a retroperitonealtumor: a case report and review

**DOI:** 10.1186/1477-7819-12-79

**Published:** 2014-03-31

**Authors:** Anca-Laura Amati, Andreas Hecker, Thilo Schwandner, Hassan Ghanem, Julia Holler, Martin Reichert, Winfried Padberg

**Affiliations:** 1 Department of General and Thoracic Surgery, University Hospital of Giessen, Rudolf-Buchheim-Street 7, 35392 Giessen, Germany

**Keywords:** Hemangioma, Mesentery, Gastrointestinal

## Abstract

Hemangiomas of the gastrointestinal tract and mesentery are uncommon benign vascular lesions. While spontaneous bleeding is the hallmark of the gastrointestinal tumor variant, clinical signs of mesenteric hemangiomas are mostly unspecific. Despite the increasing imaging quality of computerized tomography (CT), in most cases the final diagnosis is established through surgery and histopathologic analysis of a macrobiopsy.

We present a case report of a 20-year-old female patient who was admitted with progressive abdominal distension and suffered from persistent abdominal pain for 3 months. A large retroperitoneal tumor mass was detected on the CT scan. Due to radiographic signs of an intraabdominal liposarcoma, an explorative laparotomy was performed revealing a large hemangioma originating from the mesosigmoid.

Although rare, gastrointestinal hemangiomas should be kept in mind by oncological visceral surgeons as one differential diagnosis of large intraabdominal tumorous masses, especially in young adults.

## Background

Hemangiomas are benign tumor lesions, defined as vascular hamartomas of mesodermal origin [1-5]. Hemangiomas of the gastrointestinal tract are extremely rare. Those of mesenteric origin are even more seldom encountered [2]. They can occur as either single or multiple lesions. In the latter case an association to similar neoplasms at other locations is possible and can be caused by syndromes such as Osler-Weber-Rendu disease, Maffucci syndrome, Klippel-Trénaunay syndrome or the congenital blue rubber bleb nevus syndrome [6-8].

The symptoms of hemangiomas depend on the localization of the primary tumor. Nevertheless the most common primary manifestation is spontaneous bleeding [9,10]. In case of a gastrointestinal (intraluminal) localization, insidious until massive, life-threatening gastrointestinal bleeding can occur, whereas bleeding of meseteric hemangiomas leads to free intraabdominal fluid accumulation (hematoperitoneum).

Complete surgical tumor resection is the gold standard for the treatment of mesenterial hemangiomas. After complete removal of this tumor a recurrence has not been reported so far. Minimally invasive surgery is, of course, preferred to open surgery, but carries a risk of tumor bleeding, especially in cases of huge intraabdominal masses.

The purpose of this article is to highlight the clinical, radiological and histological characteristics of gastrointestinal and mesenteric hemangiomas, which are important differential diagnoses for large intraabdominal tumor masses.

## Case report

A 20-year old female patient was admitted to the University Hospital of Giessen, Giessen, Germany, with a progressive abdominal distention and increasing diffuse abdominal pain. The patient negated nausea, vomiting or any other significant constitutional symptom. The patient had no history of previous abdominal surgery or trauma and had never been in a hospital before.

Physical examination revealed a severe, central distention of the abdomen, with diffuse pain caused by digital pressure, but without any signs of peritonism. A large tumor mass was palpable extending to the whole lower abdomen on both sides. There were no abnormal findings in the routine laboratory tests. Measurement of tumor markers revealed normal levels of alpha-fetoprotein (1.8 μg/L), beta-human chorionic gonadotropin (<2.0 IU/L), β2-microglobulin (1.2 mg/L) and lactate dehydrogenase (129 IU/L). The ultrasound examination revealed an enormous tumor mass of the lower abdomen with an inhomogeneous echo pattern.

An abdominal computerized tomography (CT)scan with application of both oral and intravenous contrastmedium showed a large space-occupying intraabdominal tumor originating from the retroperitoneum, with regular borders and contrast-enhanced septation. The tumor size was approximately 12 cm x 26 cm x 28 cm. The tumor displaced the intestine and caused a compression of the bladder, without any signs of infiltration.

**Figure 1:**
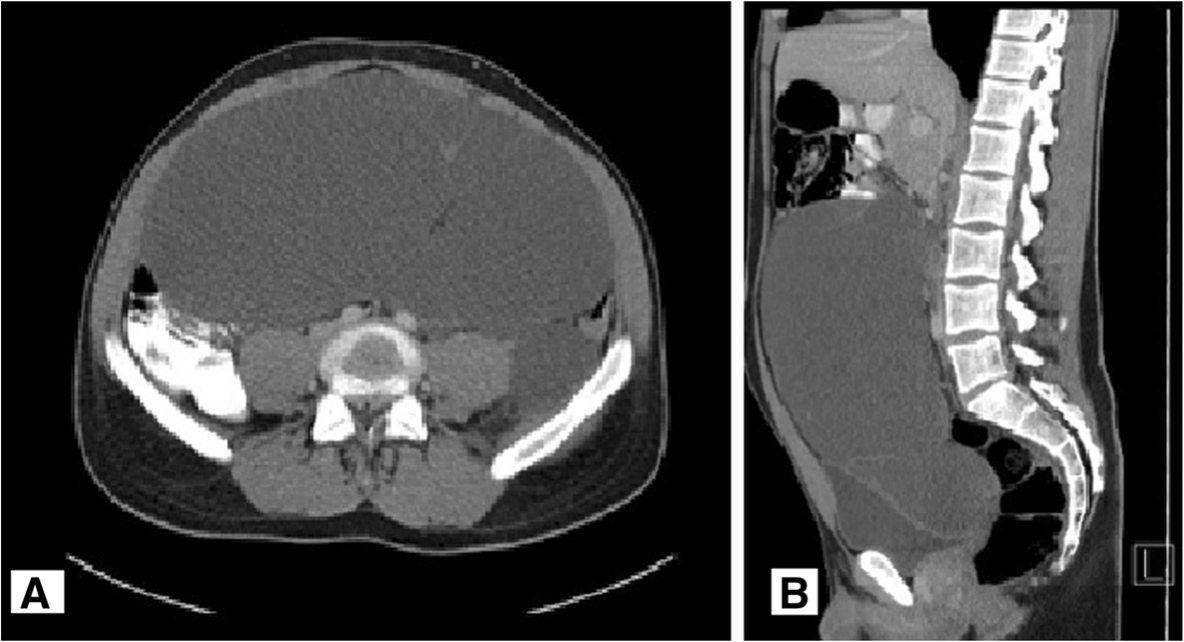
**Oral and intravenous contrast-enhanced computed tomography (CT) scans. (A)** Transverse and **(B)** sagittal imagesshowing a large abdominal tumorous mass.

The tumor extended from the level of the pancreatic head into the pelvis. There were no signs of ascites or further lesions of the intraabdominal organs (Figure 1).

**Figure 2:**
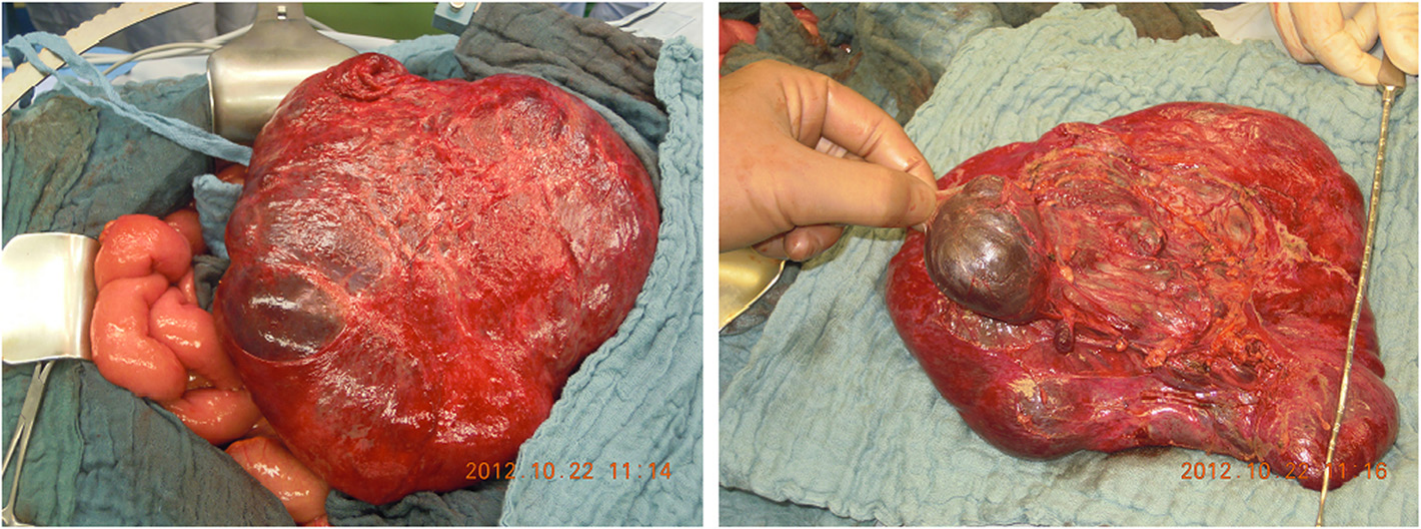
Intraoperative images of the tumorous mass originating from the mesosigmoid.

Surgical removal of the mass was indicated and an exploratory laparotomy was performed. The exploration of the peritoneal cavity revealed a large mass originating from the mesosigmoid, consisting of both solid and cystic components. The tumor was divided from the retroperitoneum by a tumor capsule (Figure 2). Both arterial and venous blood supply derived from the inferior mesenteric vessels. There were no signs of infiltration of the structures nearby the tumor. After removal of the tumor the perfusion of the sigmoid seemed to be altered. Inspection of the sigmoid showed signs of diminished vascular perfusion such as decreased pulses in the mesosigmoid and macroscopic signs of ischemia (color). A resection of the sigmoid was necessary and performed without any complications.

The patient was discharged on the seventh postoperative day. Three months of follow-up was uneventful.

**Figure 3:**
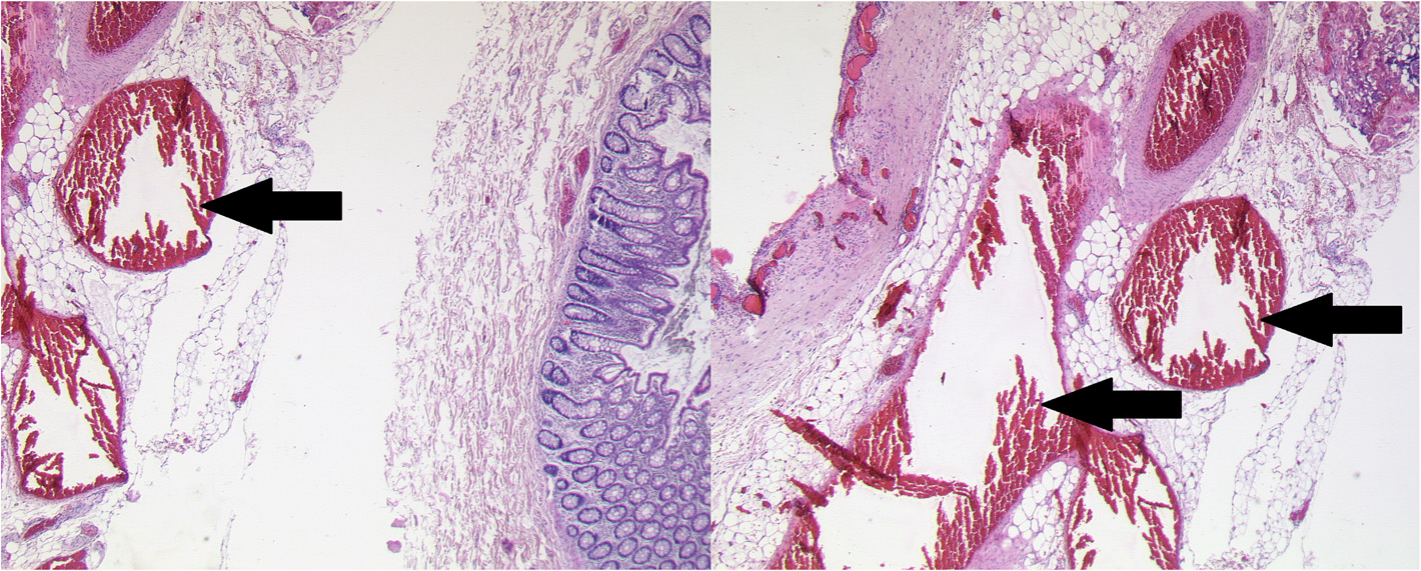
H&E staining of the resected specimen revealed characteristic dilated blood vessels (arrows) (100xmagnification).

Histopathologic examination of the resected specimen revealed an angiomatous malformation embedded in the fatty tissue of the mesosigmoid. It consisted of irregular arterial and venous blood vessels with characteristic cavernous-like dilatation and variable wall thickness. Further immunohistochemical examinations could exclude any lymphoma or mesenchymal tumor as differential diagnoses (Figure 3). The sigmoid colon showed no signs of dysplasia or inflammation.

## Discussion

Hemangiomas are uncommon hamartomatous lesions that originate from the embryonic sequestrations of mesodermal tissue and can be found in any organ [1-5]. While intestinal hemangiomas are well-known and have previously been described in literature, a cavernous hemangioma of the mesenterium remains an extremely rare tumor [2]. Additional file 1: Table S1 provides a summary of the single-case reports published in the literature. Typically hemangiomas are of benign dignity and without any potential of a malignant transformation, besides angiomatous lesions in Maffucci syndrome [6,11].

Gastrointestinal hemangiomas can occur in any age group, but are more common in young adults, often in the third decade of life [1,3,4]. No definite sex predilection has been identified [1,2].

Hemangiomas are classified histologically and named according to their major components. There are three principal types: capillary, cavernous and mixed. The cavernous variant is the most common [9].

Cavernous hemangiomas are bluish purple, soft, compressible lesions consisting of large blood-filled spaces or sinuses lined by single or multiple layers of endothelial cells histologically. Degenerative changes such as hyalinization and focal calcification develop as a consequence of thrombosis within sinuses, caused by perivascular inflammation and stasis of blood flow [10]. Calcification may over time form phleboliths which represent an important diagnostic feature, seen in 26 to 50% of affected adult patients [12]. Although no phleboliths were detected in the preoperative imaging (abdominal radiograph and CTscan) in the case presented, the histological specimen showed signs of perivascular inflammation as well as calcification next to the typical dilated blood vessels.

Depending on the tumor localization, various manifestations and symptoms have been reported in the literature. In cases of gastrointestinal localization, 80% of patients present with symptoms such as bleeding or mechanical bowel obstruction [9,10]. Hemorrhage in association with cavernous hemangiomas is usually of sudden onset and may present as either hematemesis or melena. In cases of chronic, repetitive bleeding, anemia can be the main symptom. Intussusception due to polypoid hemangiomas or bowel perforation are rare [13,14].

Bleeding of extraluminal hemangiomas, therein hemangiomas of the mesenterium or omentum, can cause a hematoperitoneum. Abdominal pain and progressive distention are the major complaints of these patients [13-15]. In the case described, the patient presented with abdominal pain and distention. There were no signs of free intraabdominal fluid in the ultrasound performed on admission or the CTscan performed the following day. The symptoms were most likely caused by the massive extent of the lesion. Obviously the rapid abdominal distension (over the course of 3 weeks) was caused by an intratumoral hemorrhage. The latter could be confirmed by histological examination of the resected specimen.

Gastrointestinal bleeding, palpable abdominal mass as well as asymptomatic cases of mesenteric hemangiomas have also been described [2,10,16,17]. Reports reveal that cavernous hemangiomas are not associated with other abnormalities. However, a rare case of a cavernous hemangioma of the intestine and mesenterium associated with Kasabach-Merritt syndrome (also known as hemangioma thrombocytopenia syndrome) has been reported [18]. On the other hand, gastrointestinal hemangiomatosis, manifesting as a diffuse infiltration of the intestinal wall, the mesenterium and occasionally the retroperitoneum, is associated with a number of syndromes such as blue rubber bleb nevus syndrome, Klippel-Trénaunay-Weber syndrome, Maffucci syndrome, diffuse neonatal hemangiomatosis and Proteus syndrome [6-8]. Maffucci syndrome is the only described condition in which a malignant transformation of an angiomatous lesion has been observed [11].

Despite advanced imaging techniques, gastrointestinal and mesenteric hemangiomas are often misdiagnosed. Due to unspecific symptoms, an average duration of 19 years from symptom onset until the final correct diagnosis is described in the literature [19].

On plain radiograph and barium studies, multiplephleboliths, especially in atypical position, should indicate a hemangioma as a possible diagnosis [20,21]. Other signs such as mass effects with intestinal displacement as well as mucosal irregularity or narrowing of the intestinal lumen may also be encountered. Endoscopic examinations are useful in case of upper gastrointestinal tract or colon involvement but carry the risk of massive bleeding.

Ultrasound examination usually reveals a solid mass of mixed echogenicity, whereas CTscan additionally provides information concerning the tumor extent, multiplicity, vascularization and involvement of the intestine or other intraabdominal or retroperitoneal structures [22]. Due to a lack of specific signs such as phleboliths, preoperative imaging in the case presented failed to establish the correct diagnosis. In contrast, the lipomatous character of the tumor on the CTscan led to the radiological diagnosis of a liposarcoma. Nevertheless the CTscan was useful to analyze the extent of the tumor and to exclude any involvement of other organs and structures.

Mesenteric angiography can be a useful tool in cases of acute bleeding, providing both a precise tumor localization and the possibility of an interventional ablation of the arterial inflow. The technique requires an adequate rate of bleeding (0.5 mL/min) to be successful. In patients with diffuse hemangiomatosis of the mesentery, where a bleeding out of a single vessel cannot be identified, a selective mesenteric angiography can be useful in determining the exact localization of the bleeding as well as the extent of resection to be undertaken [23]. Magnetic resonance imaging (MRI) of cavernous hemangioma typically shows a uniform high signal intensity on T2-weighted images and is more clearly depicted with fat suppression. Degenerative changes such as fibrosis and calcification lead to a heterogeneous signal intensity on T2-weighted images, whereas intratumoral hemorrhage may show low intensity, making hemangiomas hard to differentiate from other solid mesenchymal tumors such as fibroma or leiomyoma [2,19,21,24,25].

The treatment of cavernous hemangioma of the gastrointestinal tract or mesentery is either open or laparoscopic surgical excision. A minimally invasive laparoscopic approach to diagnose the tumor dignity by surgical macrobiopsy could be considered as the gold standard for intraabdominal tumors of unknown origin and entity. Nevertheless, we preferred open surgery due to the tumor size and bleeding risk. Nonoperative techniques such as sclerotherapy, cryosurgery or interventional angiography usually result in symptom recurrence and are therefore temporary solutions. Recurrence after complete resection has not been reported [3,25].

## Conclusion

Hemangiomasof the mesentery, although extremely rare, should be considered as a differential diagnosis in patients presenting with episodes of abdominal pain and imaging showing large intraabdominal tumorous masses. Despite modern radiographic imaging techniques the differential diagnosis of a soft tissue sarcoma is more common and might lead to a tumor biopsy. Bearing in mind that a percutaneous biopsy of hemangiomas could have aggravating consequences, exploratory laparotomy or laparoscopy should be indicated, if imaging techniques leave place for doubt.

## Consent

Written informed consent was obtained from the patient for the publication of this report and any accompanying images.

## Abbreviations

CT: Computerized tomography; HE: Hematoxylin and eosin; MRI: Magnetic resonance imaging.

## Competing interests

The authors declare that they have no competing interests.

## Authors’ contributions

AA and AH wrote the manuscript. TS, JH, HG and MR performed histological stainings and radiographic imaging. WP is the responsible chief of the department. Surgical treatment of the patient was performed by him. All authors read and approved the final manuscript.

## Supplementary Material

Additional file 1: Table S1 Summary of the single-case reports on mesenteric hemangiomas published in the literature [2,12,15-17,22,26-36]. Click here for file
